# Validity of Cardiovascular Risk Prediction Models in Kidney Transplant Recipients

**DOI:** 10.1155/2014/750579

**Published:** 2014-04-08

**Authors:** Holly Mansell, Samuel Alan Stewart, Ahmed Shoker

**Affiliations:** ^1^College of Pharmacy and Nutrition, University of Saskatchewan, Saskatoon, SK, Canada S7N 5E5; ^2^Department of Medicine, University of Saskatchewan, Saskatoon, SK, Canada S7N 5E5; ^3^Department of Medicine, Division of Nephrology, College of Medicine, University of Saskatchewan, Saskatoon, SK, Canada S7N 5E5

## Abstract

*Background.* Predicting cardiovascular risk is of great interest in renal transplant recipients since cardiovascular disease is the leading cause of mortality. *Objective.* To conduct a systematic review to assess the validity of cardiovascular risk prediction models in this population. *Methods.* Five databases were searched (MEDLINE, EMBASE, SCOPUS, CINAHL, and Web of Science) and cohort studies with at least one year of follow-up were included. Variables that described population characteristics, study design, and prognostic performance were extracted. The Quality in Prognostic Studies (QUIPS) tool was used to evaluate bias. *Results.* Seven studies met the criteria for inclusion, of which, five investigated the Framingham risk score and three used a transplant-specific model. Sample sizes ranged from 344 to 23,575, and three studies lacked sufficient event rates to confidently reach conclusion. Four studies reported discrimination (as measured by *c*-statistic), which ranged from 0.701 to 0.75, while only one risk model was both internally and externally validated. *Conclusion.* The Framingham has underestimated cardiovascular events in renal transplant recipients, but these studies have not been robust. A risk prediction model has been externally validated at least on one occasion, but comprehensive validation in multiple cohorts and impact analysis are recommended before widespread clinical application is advocated.

## 1. Introduction

Cardiovascular disease (CVD) is the leading cause of morbidity and mortality in renal transplant recipients (RTR). Accounting for more than 30% of deaths [1, 2], the risk of a cardiovascular event (CVE) is greatly increased in this population when compared to the general public [3, 4].

Traditional risk factors such as diabetes, hypertension, and dyslipidemia partially explain why the incidence of CVD in this group is so high, yet a combination of other transplant-specific factors significantly impact risk [3, 5]. They include pretransplantation exposure to chronic kidney disease- (CKD-) related risk factors, allograft dysfunction, and chronic exposure to immunosuppressive agents [6]. Other nontraditional markers of inflammation such as homocysteine and C-reactive protein have also emerged as risk factors for CVD in RTR [7, 8]; thus, the aetiology is far more complex than what is seen in the general population.

Risk prediction models are used in the general population to forecast cardiovascular events (CVE) and to tailor preventative therapy, yet their validity remains questionable in transplant populations. Currently, the Framingham risk score (FRS) [9] is used to predict the risk of developing a coronary event within the following 10 years, but it is generally accepted that this model underestimates CVD risk in RTR [10, 11]. Despite its limitations, the FRS calculator has been used loosely to calculate CVD risk and to measure CVE in RTR outcomes [12–15], due to its simplicity and accessibility. Other nontransplant based prediction models include Reynolds Risk Score, the Prospective Cardiovascular Münster Heart Study (PROCAM), the Systemic Coronary Risk Evaluation system (SCORE), and the QRISK 1 and 2 [16–20]. Recently, risk calculators for major adverse cardiac events (MACE) and mortality have been developed in RTR [21].

Given the high CVD burden in this population, along with the availability of multiple risk calculators, we conducted this systematic review. Our aim was to assess the use, validity, and limitations of CVD risk scoring systems in RTR, as no previous group, to our knowledge, had accomplished this task.

## 2. Materials and Methods

The protocol for this review was registered in PROSPERO, an international database of prospectively registered systematic reviews in health and social care. It is accessible at http://www.crd.york.ac.uk/ under the registration number CRD42013004606.

### 2.1. Data Sources and Searches

A systematic review was performed, and the databases searched included MEDLINE via OVID SP (1950 to present with daily update), EMBASE via OVID SP (1947 to present with daily update), CINAHL via EBSCO, SCOPUS, and Web of Science (1900 to present). Our search included the terms (1) cardiovascular, (2) prediction rule (Framingham or PROCAM or ASSIGN or QRISK1 or QRISK2 or SCORE or Reynolds Score or risk assessment or risk score or prediction rule), and (3) kidney or renal transplant. The complete search strategy is available (see Supplementary Material available online at http://dx.doi.org/10.1155/2014/750579). Duplicate records were removed via electronic software (Ref-Works software, ProQuest LLC, Ann Arbor, MI), and two independent reviewers screened the remaining abstracts. Additional studies were sought out by hand searching through the reference lists of all included articles. Articles unrelated to the focus of the project were excluded. Articles deemed as potentially includable by at least one reviewer were then read in full by both authors and disagreements were resolved by discussion.

### 2.2. Study Selection and Data Extraction

Studies were included if they were longitudinal cohort studies involving RTR, with at least 100 participants and at least one year follow-up. Cohort studies could be either prospective or retrospective, with prospective data collection. Abstracts from conference proceedings were excluded. The following variables were extracted from each study: population characteristics, study setting, number of participants, risk scoring system, inclusion criteria, primary outcome, number of events, and length of follow-up. Prognostic performance was measured by area under the receiver operating characteristic curve (*c*-statistic), ratio of predicted/observed event rates, sensitivity and specificity, and diagnostic accuracy. Similar to a recent review on risk prediction models in chronic kidney disease [22], methodological quality was assessed using the parameters outlined by Tangri et al. [22] based on the reporting of discrimination and calibration of models, along with model fit statistics and reclassification reports. Bias was assessed using the approach recommended by Hayden and colleagues [23, 24]. The Quality in Prognostic Studies (QUIPS) tool involves using evaluation criteria consisting of 6 categories: study participation (sampling bias), study attrition (attrition bias), prognostic factor measurement, outcome measurement (ascertainment bias), confounding measurement and account, and analysis and reporting (Table 1).

## 3. Results

**Figure 1:**
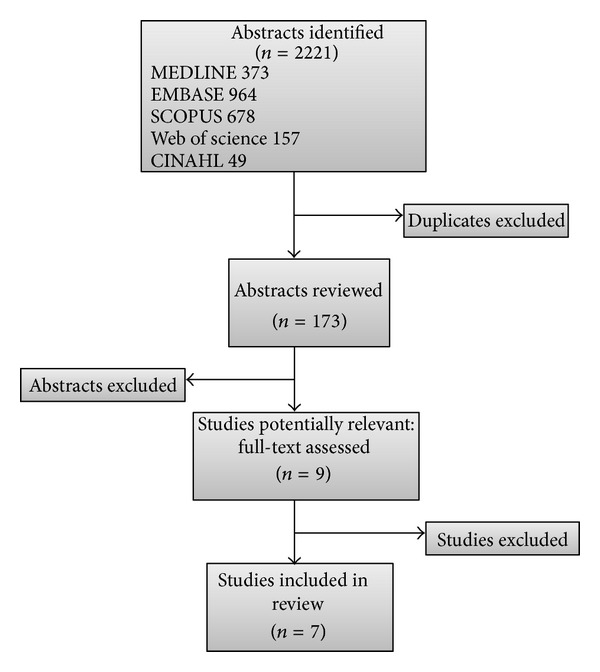
Flow chart describing study selection process.

Of the 173 titles and abstracts reviewed, nine studies were identified.  Figure 1 illustrates the process of our search strategy and number of studies. Seven studies in total were included in the systematic review, with five studies involving the FRS and two studies using the MACE calculator for renal transplant recipients. The study size ranged from 344 to 23,575 and in total consisted of 30,891 participants.  Table 2 represents a summary table of the studies included in our review, while Table 3 describes the studies excluded and why.  Table 4 presents the risk of bias and model evaluation results. Of the seven studies, only one reported model fit statistics, and three did not report either discrimination or calibration results.

Using the QUIPS method [24], we evaluated bias across 6 dimensions listed in Table 3. As the table demonstrates, there is a potential for bias in all the papers, though only 1 [11] hassignificant bias. The papers were generally good at reporting study populations but there was incomplete reporting of study attrition, particularly the missing values. The descriptions of the outcome variable were well identified, though varied between papers. The description of the predictors was weak, and notably there was significant variation in confounding variables included in the models. The analyses tended to be accurately reported but brief, with little discussion of Tangri's model components.

### 3.1. Brief Discussion of Selected Studies

Kasiske and colleagues [25] were first to report on the predictive value of the FRS equation in 1500 renal transplant recipients using a Cox proportional-hazards model. The study excluded patients experiencing IHD within one year of transplant, which permitted the authors to study the relationship between posttransplant conditions, but resulted in the exclusion of 107 patients. A follow-up period of only one year is a limitation. The authors deduced that FRS predicted ischemic heart disease (IHD) with a relative risk of 1.28 (95% CI 1.20–1.40; *P* < 0.001) but underestimated risk in RTR. This underestimation was most notable in patients with diabetes mellitus and to a lesser extent with age and cigarette smoking. The study was not designed to validate the FRS in this population, but rather the objective was to compare observed-versus-expected incidence of IHD based on relationships of risk factors and IDH in FRS. As such, more robust measures of performance such as discrimination, calibration, or even odd ratios were not presented. Furthermore, significant differences were observed between the development and the validation cohort. The outcome of IHD was defined by MI or coronary revascularization or death and the sample population intentionally excluded angina pectoris and CHF.

Ducloux and colleagues [10] prospectively assessed the relevance of the FRS in 344 stable transplants in France. The FRS accurately predicted CVD risk in the low-risk RTR but underestimated CVE in the high-risk group. Overall, the observed-versus-expected incidence of predicted risk was 1.28 (CI 0.20–1.040; *P* < 0.0001). It is noteworthy that several other retrospective studies have concluded that the FRS overestimates CV risk in the French general population, so perhaps the ability of the FRS to accurately predict events in the low-risk population was a reflection of the overestimation of events previously observed in French cohorts [26]. Hypertension was not significantly associated with CVD, leading the authors to question sample size and follow-up. Furthermore, only 27 cardiovascular events in total were observed. It has been suggested that a validation sample for prediction rules should consist of a minimum of 100 events and 100 nonevents to detect substantial changes in accuracy [27].

Kiberd and panek [28] determined the relevance of FRS in a cohort of 540 RTR. The authors used a more inclusive definition of MACE as the primary outcome, including cerebral vascular events and other significant events like CHF, significant rhythm disturbances, and peripheral vascular disease, in addition to MI, coronary revascularization, and death. Rates per 100-patient years were 1.79 (*n* = 38) for cardiac and 0.78 (*n* = 16) for stroke events, with FRS underestimating observed cardiac events but not stroke. The ratio of observed-to-predicted cardiac event ratios for the entire cohort was 1.64 (95% CI 1.19–2.94) and* c-*statistics were 0.646 (95% CI 0.539–0.720, *P* = 0.003) for MACE, 0.713 (95% CI 0.598–0.827, *P* = 0.004) for stroke, and 0.701 (95% CI 0.65–0.752, *P* < 0.001) for all events. The largest overestimation occurred in patients aged 45–60. Again, a major weakness with this work was the small number of events.

A more recent attempt to quantify predictive value of the FRS was undertaken by Silver and colleagues [11]. A database review of patients who underwent transplant from 1998 to 2008 resulted in an underestimate of CV events in an ethnically diverse cohort from Toronto, Canada. The actual-to-predicted event ratio in this group ranged from 1.2 to 8.4 (*P* < 0.001) between the various subgroups analyzed, with the highest underestimation occurring in RTR with diabetes, smoking, or a high FRS. This study also investigated novel risk markers including C-reactive protein, uric acid, and urine albumin-to-creatinine ratio but showed that only risk scores equivalent to or greater than 10% (hazard ratio 2.313, 95% CI 1.49–3.58, *P* < 0.002) and eGFR less than 50 mL/min (hazard ratio 2.291, 95% CI 1.49–3.58, *P* = 0.034) predicted MACE in the multivariate analysis. Novel risk factors did not improve the predictive ability. Patient characteristics were not well described in that manuscript, leading us to question the impact of ethnic diversity. The original Framingham cohort consisted of predominantly white, middle-class Americans, and an underestimation of cardiovascular risk has resulted from using the scoring system in several other populations including Asian, Native American, and Indian patients [29–31]. The authors do state that 58% of the cohort was white, however, given that nearly half of Toronto's population is a visible minority [32], ethnicity is a potential confounding factor. Further, the primary outcome in this study was MACE, defined by fatal or nonfatal MI, coronary revascularization, or cardiac death, yet a much more inclusive definition was chosen to define patient history of pretransplant cardiac disease. The authors argued that this outcome did not include angina or silent MI, to correspond with endpoints used in current clinical trials. Again, with only 89 events observed in this population, one may question the statistical power [27].

The Patient Outcomes in Renal Transplantation (PORT) study [33] was the first attempt to use a large multicenter database to develop a CVD risk prediction model specifically for RTR. Of the 88 transplant centers contacted worldwide, 14 centers (16%) from North America, Northern and Southern Europe, and the Pacific Rim provided useable data, amounting to a total study sample of 23,575. Participating centers submitted data on a number of recipients, donor elements, and transplant procedure elements, and the patients were randomized to either the development subset (70%) or the validation subset (30%). From Cox proportional hazard analyses, three CVD risk-prediction models were generated. The first model predicted risk within the first year posttransplant using variables available at the time of transplant (including age, sex, history of diabetes, history of cancer, number of comorbid CVD conditions, donor type, BMI, and years end stage renal disease to transplant) and performed with a time-dependent* c-*statistic ranging from 0.80 to 0.85. The second model also predicted CHD risk within the first year posttransplant but used data from the first week of posttransplant and had a* c-*statistic range of 0.73–0.83. This model, which was conditional on the seven-day survival without a CVE, included age, sex, diabetes, number of cardiovascular comorbid conditions, BMI, years from first dialysis, and delayed graft function as variables. The third model predicted CHD within three years of a clinic visit with 1–5 years posttransplant and performed with a* c-*statistic of 0.73–0.80. Twelve variables were included in this model (age, sex, race, most recent panel reactive antibodies at time of transplant, year from first ESRD treatment to transplant, acute rejections in prior year, posttransplant lymphoproliferative disorder, diabetes, eGFR, number of cardiovascular comorbid conditions, posttransplant CVD or PVD events, and delayed graft function). None of the PORT models to date have been externally validated.

In a subset of the PORT patients, the PORT model performed better than the FRS. It was also reported that the FRS variables did not significantly improve risk prediction (likelihood ratio test, *P* = 0.0937).

Soveri and colleagues [21] developed a cardiovascular risk and mortality prediction tool from the ALERT multicenter clinical trial [34]. The population was randomly divided into an assessment sample (67%) and a test sample (33%) and variable selection was accomplished with a backward stepwise Cox regression. Risk was calculated for individual patients in the assessment sample (*n* = 1329) with the prognostic index and the probability of survival per patient, and the equation was validated with the test sample (*n* = 701). The MACE model included age, previous CHD, diabetes, LDL, SCr, number of transplants, and smoking, and discrimination was reported with a* c-*statistic of 0.738 in the assessment sample and 0.740 in the test sample. Calibration of this model was reported as good with the Hosmer-Lemeshow test of 11.47 and a degree of freedom (df) of 8 (*P* = 0.245), indicating that the model fit was acceptable. Participants in the ALERT trial consisted of renal and combined renal/pancreas transplants, at least 6 months posttransplant, and received cyclosporine-based immunosuppression. The generalizability of the prediction rule, however, will be limited by the inclusion criteria of the clinical trial, and the authors acknowledge that high-risk patients may have been excluded from the study and care should be taken when applying this risk prediction method to patients on risk extremes.

Soveri and colleagues [35] performed a follow-up study with the aim of externally validating the equations using data from the PORT population. There were a total of five centers reporting on 4,146 living recipients with a functioning graft at the end of one year. Complete reporting for all necessary variables was available for 72% resulting in a validation population of 2,967 from Europe and the United States. Discrimination was reported by a* c-*statistic of 0.740 and the Hosmer-Lemeshow test for calibration indicated a significant lack of fit *χ* ^2^ = 19.49 with 8 degrees of freedom, *P* = 0.01), underestimating CV risk in deciles 5 and 9.

## 4. Discussion

This review identified six studies (seven published papers) attempting to create, validate, or improve on CVD risk prediction models. The FRS is arguably the most common risk prediction model used in the general population and five studies [10, 11, 25, 28, 33] investigated its validity in kidney transplant populations.

Prediction rules generated from training samples commonly show a reduced accuracy when validated in new cohorts [36, 37]. As explained by Tolle and colleagues [38], a main attributing factor is the difference between the training and validation populations, which poses a serious challenge to applying the FRS to the transplant populations. Our review identified several differences between the original Framingham population and the transplant cohorts, including discrepancies in the definition of the outcome variable (i.e., how CHD was defined), differences in predictor definition (e.g., smoking and diabetes), diversity between patient characteristics (e.g., age, ethnicity, clinical stability, or patient health), and variability in event rates. In addition, three of the five Framingham transplant studies [10, 11, 28] consisted of fewer than 100 events, so it is questionable whether or not these studies had adequate statistical power [27].

Keeping in mind the limitations of updating prediction rules in a new population, it is not surprising that all of these studies found that the FRS underestimated events in the transplant cohorts compared to the general population [3, 4]. We believe that the addition of several unique transplant-related factors may account for this difference. Nontraditional factors have shown to independently predict cardiovascular disease in this population such as albuminuria, anemia and graft rejection [39], time on dialysis before transplantation [40], donor history of hypertension [41], immunosuppressive regimen [42], quality of allograft function [43], elevated homocysteine [44], and C-reactive protein [8]. Some authors have attempted to update the FRS with more transplant specific variables (such as C-reactive protein, homocysteine, uric acid, and albumin-creatinine ratio) [10, 11], but these studies were not robust enough to test this hypothesis or derive a predictive formula. Of interest and similar to the transplant studies, the FRS has underestimated cardiovascular events in chronic kidney disease [45]. This is not surprising since GFR has been shown to be an independent predictor for CVD [46, 47], and the FRS does not account for this variable. Further evidence to support the importance of transplant specific variables is illustrated in the PORT study [33]. In these equations, novel risk factors such as delayed graft function, acute rejection, and eGFR predicted cardiovascular disease reasonably well, with the FRS score adding little predictive value.

The use of new CVD risk calculators results in models which require additional external validation [48]. Pita-Fernandez and colleagues [49] plan to examine four CV risk prediction models calculated at the time of transplant: the FRS, the European Systematic Coronary Risk Evaluation (SCORE) equation, the REGICOR (REgistre Gironí del COR (Gerona Heart Registry)), and the DORICA (Dyslipidemia, Obesity, and Cardiovascular Risk) (the latter two are adaptations from the Framingham equation for Spanish population characteristics). The authors hope to apply these models to compare several transplant specific variables including donor and recipient characteristics, chronic kidney disease-related risk factors, pretransplant and posttransplant CV risk, routine biochemistry, immunosuppressive, antihypertensive, and lipid-lowering therapy. The results of this analysis are not yet published.

Model performance is important, but alone it does not translate into widespread clinical acceptance [50]. Impact studies are necessary to quantify the effect of using the model on doctor's behavior, patient outcome, or cost effectiveness and can determine whether the use of a model is better than usual care [51]. Impact studies offer the further advantage of investigating factors that may affect implementation of a prognostic model, such as the acceptability of the prognostic model to clinicians and ease of use [51]. Several practical barriers may prevent widespread use of models and the user-friendliness should be taken into account when developing the rule. While Soveri and colleagues [21, 35] aimed to demonstrate the application of the prediction model (in two clinical trials), none of the reviewed studies highlighted the importance of model impact assessment. The PORT prediction models [33] performed reasonably well and allowed the clinician to predict CVD risk at clinically important time points posttransplant. Their application in practice, however, may seem cumbersome and time consuming, given that clinicians will need to choose between 3 risk-prediction models and assess a large number of variables (8, 7, or 12) dependent on the applicable model.

There are limitations to our work. As with any systematic review, conclusions are dependent on the quality and availability of studies. While our review identified seven reports acceptable for inclusion, the quality was not sufficient to perform a meta-analysis or perform a forest plot, due to the varying definitions of outcomes and inconsistent use of prognostic factors. While our search strategy consisted of five reputable databases, we did not search abstracts from conference proceedings; hence, the possibility of publication bias deserves mention. Language bias may be present since our search strategy included articles in English. Our search terms specifically included the names of well-known CVD risk scoring systems (FRS, or PROCAM, or ASSIGN, or QRISK1, or QRISK2, or SCORE, or Reynolds Score) but did not include less publicized scoring methods or those used in other countries such as the DORICA, although it is likely that such studies would have been discovered under the search for “risk score*.” Further, we limited our review to include “cardiovascular risk” rather than including “mortality risk,” rationalizing that mortality in transplant recipients may also be attributed to causes other than cardiovascular disease (such as rejection or infection). We assessed bias based on the method suggested by Hayden and colleagues [23, 24], as, to date, no other validated method exists for assessing bias in predictive studies.

To summarize, the FRS has consistently been found to underestimate CVD risk in RTR, but in general, these studies have not been robust. It is likely that too much diversity exists between the general population and RTR to accurately translate risk prediction from one group to another.

Studies that have moved beyond the FRS have found improved prognostic powers, but there is still more room for improvement. Soveri and colleagues have developed a seven-year model, which showed acceptable internal discrimination and calibration, but external validation revealed that further refinements may be necessary to improve calibration. Comprehensive validation in multiple cohorts and impact analysis is recommended before widespread application is advocated. Adoption into practice will ultimately depend on clinician acceptance.

## Supplementary Material

Five databases were searched for this systematic review, including MEDLINE via OVID SP (1950 to present with daily update), EMBASE via OVID SP (1947 to present with daily update), CIHNAL via EBSCO, SCOPUS, and Web of Science (1900 to present). The complete search strategy for MEDLINE is listed below. Search strategies were modified appropriately with the assistance of a medical librarian for EMBASE, SCOPUS, and Web of Science.

## Figures and Tables

**Table 1:** Criteria for determining risk of bias (adapted from the QUIPS tool*).

Potential bias	Areas to be considered
*Study participation* The study sample adequately represents the population of interest	(i) Adequate participation in the study by eligible persons (ii) Description of the source population or population of interest (iii) Description of the baseline study sample (iv) Adequate description of sampling time frame and recruitment (v) Adequate description of the period and place of recruitment (vi) Adequate description of inclusion and exclusion criteria

*Study attrition* The study data available (i.e., participants not lost to follow-up) adequately represent the study sample	(i) Adequate response rate for study participants (ii) Description of attempts to collect information on patients who dropped out (iii) Reasons for loss to follow-up are provided (iv) Adequate description of participants lost to follow-up (v) There are no important differences between participants who completed the study and those who did not

*Prognostic factor measurement* The prognostic factor is measured in a similar way for all participants	(i) A clear definition or description of the PF is provided (ii) Method of PF measurement is adequately valid and reliable (iii) Continuous variables are reported or appropriate cut points are used (iv) The method and setting of measurement of PF are the same for all study participants (v) Adequate proportion of the study sample has complete data for the PF (vi) Appropriate methods of imputation are used for missing PF data

*Outcome measurement* The outcome of interest is measured in a similar way for all participants	(i) A clear definition of the outcome is provided (ii) Method of outcome measurement used is adequately valid and reliable (iii) The method and setting of outcome measurement are the same for all study participants

*Study confounding* Important potential confounding factors are appropriately accounted for	(i) All important confounders are measured (ii) Clear definitions of the important confounders measured are provided (iii) Measurement of all important confounders is adequately valid and reliable (iv) The method and setting of confounding measurement are the same for all study participants (v) Appropriate methods are used if imputation is used for missing confounder data (vi) Important potential confounders are accounted for in the study design (vii) Important potential confounders are accounted for in the analysis

*Statistical analysis and reporting* The statistical analysis is appropriate, and all primary outcomes are reported	(i) Sufficient presentation of data to assess the adequacy of the analytic strategy (ii) Strategy for model building is appropriate and is based on a conceptual framework or model (iii) The selected statistical model is adequate for the design of the study (v) There is no selective reporting of results

*Adapted from reference [24].

QUIPS: Quality in Prognosis Studies; PF: prognostic factor.

**Table 2:** Characteristics of studies included in systematic review of cardiovascular risk prediction models in renal transplant recipients.

Study	Objective	Outcome	Design	Sample size and inclusion criteria	Age/sex/events	Follow-up	Scoring system	Summary of results	Methodology quality
Kasiske et al. [25] 2000	To compare observed and expected incidence of IHD based on relationships of risk factors and IDH in FRS	IHD defined by MI or coronary revascularization or death due to IHD	Retrospective cohort study (United States)	*n* = 1124 (excluding IHD pretransplant (*n* = 107) and within the 1st year (*n* = 43), as well as RTR that did not survive >1 year with a functioning graft (*n* = 236)) *n* = 1500 (including IHD pre- or <1 year posttransplant)	40.1 ± 12.8 years (at time of transplant) 56.4% male 123 events excluding those with IHD within the 1st year posttransplant	At least 12 months Chart records reviewed from 1963 to 1997	Framingham [11] (variables included age, gender, blood pressure, HDL cholesterol, diabetes, current smoking)	FRS predicted IHD but underpredicted risk in some populations, especially diabetes; overall actual to predicted risk of IHD was 1.28 (CI 1.20–1.40; *P* < 0.0001)	Sample population intentionally excluded angina pectoris and CHF, in contrast to the original Framingham cohort; IHD pretransplant and within the 1st year were also excluded; robust measures of performance were not included in this analysis.

Ducloux et al. [10] 2004	To determine incidence and risk factors for IHD and assess relevance of FRS in RTR	Coronary heart disease defined by document MI or coronary revascularization or typical angina	Prospective cohort study (France)	*n* = 344 RTR free of vascular disease, >12 months posttransplant, without acute rejection or Scr > 400 umol/L	51 ± 13.7 years 63% male (*n* = 217) 27 events in total	72 ± 14 months	Framingham [11] (C-reactive protein and serum homocysteine also investigated)	Although the FRS predicted IHD in low-risk patients, it was underpredicted in high-risk patients: observed versus expected incidences were low-FRS = 0.6% versus 0.51%; high-FRS = 6.4% versus 2.8%	Sample population not identical to FRS; hypertension not significantly associated with CVD leading the authors to question sample size and follow up; *c*-statistics not used

Kiberd and Panek [28] 2008	To assess the ability of the FRS to predict CV events	MACE defined as fatal and nonfatal MI and invasive coronary artery therapy, cerebral vascular events, and other (CHF and PVD, rhythmic)	Prospective cohort study (United States)	*n* = 540 RTR >6 months posttransplant	48 ± 12 years 59% male (*n* = 322) events: *n* = 38 for cardiac *n* = 16 for stroke *n* = 92 for all combined	4.73 years	Framingham CV and stroke score [11]	FRS underestimated CVE across the entire cohort (observed to predicted risk 1.64 CI 1.19–2.94), but more so in patients aged 45–60 with CVD or diabetes (observed to predicted risk 2.74 CI 1.7–4.24)	Small number of events and sample limited ability to validate or develop new score; study used prevalent RTR > 6 months posttransplantation versus incident patients

Israni et al. [33] 2010	PORT dataset used to identify CHD predictive risk factors to develop risk-prediction equations at clinically important time points	CHD defined as fatal or nonfatal AMI, coronary revascularization, or sudden death.	Retrospective cohort study (14 centers worldwide)	*n* = 23 575 *n* = 3880 for FRS comparison* kidney-only transplants; only centers that could report on required variables were included	18–34 years = 22% 35–49 years = 36% 50–64 years = 34% ≥65 = 8% 60% male 689 events in year 1; 669 events in years 1–5; 143 events for FRS comparison*	4.5 years (data collected from Jan 1, 1990, to 2007)	2 models were developed to predict CV risk within 1 year and 1 model to predict risk within years 1–5 posttransplant (8, 7, and 12 variables, resp.)	The 3 models performed reasonably well with a time-dependent *c*-statistic of >0.75. Transplant related factors (e.g., DGR, AR, and eGFR) could predict CVE without FRS and FRS added little predicted value	Multicenter data with large sample size; models developed had many variables (8, 7, and 12), which predicted risk at clinically important time points. The model was not externally validated.

Silver et al. [11] 2011	To quantify predictive value of FRS and determine whether novel factors could improve	MACE defined by fatal or nonfatal MI or coronary revascularization or cardiac death	Retrospective cohort study (Canada—ethnically diverse cohort)	*n* = 956 Transplanted between 1998 and 2008, with >3 months of graft function	RTR with CVE: 58.3 ± 11 years RTR without CVE: 52.3 ± 12 years RTR with CVE: 81% male RTR without CVE: 61% male 89 events in total	4.15 years	Framingham [11] (C-reactive protein, uric acid, urine albumin-creatinine ratio also investigated)	FRS underpredicted events in all subgroups (actual to predicted event ratio was 1.2–8.4; *P* < 0.0001), but most notably in RTR with a history of diabetes or smoking. GFR was only non-FRS variable of predictive value after MVA (CRP, UA, and urine ACR did not).	Definition of MACE did not include angina or silent MI yet a much more inclusive definition was used for pretransplant CV history resulting in inconsistency; ethnicity was not accounted for as a confounder; small sample size

Soveri et al. [21] 2012	ALERT (a multicenter clinical trial) dataset used to develop and validate an equation for CV risk and mortality prediction in RTR	MACE defined by cardiac death, nonfatal MI, or coronary revascularization	Retrospective cohort study (multicenter-Northern Europe and Canada)	*n* = 1329 Renal and combined renal/pancreas, >6 months posttransplant; all were on CSA based IS, none were on statins: unstable angina within prior 6 months were excluded	50 ± 10.9 years 65.5% male (*n* = 917) 165 events in total	6.7 years	A 7-year MACE and mortality calculator for RTR Variables included age, CHD, SCr, LDL, smoking, diabetes, time on dialysis, and number of transplants	A formula for a 7-year MACE and mortality prediction was developed using a 7-variable model; MACE model had a *c*-statistic of 0.738 and 0.740 in the assessment and test samples, and mortality model had a *c*-statistic of 0.734 and 0.720 in assessment and test samples, respectively	MACE prediction tool developed from population specific variables in a sufficiently sized dataset; model was internally validated and discrimination and calibration were both reported; generalizability limited to dataset inclusion criteria

Soveri et al. [35] 2013	To externally validate the 7-year MACE and mortality calculators for RTR using the PORT dataset	MACE defined by cardiac death, nonfatal MI, or coronary revascularization All patient follow-up was censored at graft loss.	Retrospective cohort study (Europe and the United States)	*n* = 2967 Kidney-only transplants; only centers that could report on required variables were included	Age and sex not reported (211 events in total)	Median follow-up with functioning graft was 4.7 years (33% had at least 7 years of follow-up)	7-year MACE and mortality calculator for RTR	MACE could be predicted with a discrimination of 0.740 but calibration indicated significant underestimation in risk in decile 5 and 9; mortality *c*-statistic was 0.721; underestimation of risk in decile 7 and overestimation in the highest risk decile	External validation of the 7-year MACE and mortality calculator for renal transplants in a large database. Some limitations with respect to model performance which can be attributed to differences in datasets.

FRS: Framingham risk score; RTR: renal transplant recipients; IHD: ischemic heart disease; MI: myocardial infarction; CHF: congestive heart failure; SCr: serum creatinine; CV: cardiovascular; CVD: cardiovascular disease; MACE: major adverse cardiovascular event; CHF: congestive heart failure; PVD: peripheral vascular disease; DGF: delayed graft function; AR: acute rejection; eGFR: estimated glomerular filtration rate; CRP: C-reactive protein; UA: uric acid; urine ACR: urine albumin-to-creatinine ratio; MVA: multivariate analysis; ALERT: Assessment of Lescol in Renal Transplantation; CSA: cyclosporine; IS: immunosuppression; CHD: coronary heart disease; PORT: patient outcomes in renal transplant; LDL: low density lipoprotein cholesterol.

**Table 3:** Studies excluded from the systematic review and reason for exclusion.

Study	Objective	Outcome	Design	Sample size and inclusion criteria	Follow-up	Scoring system	Summary of results	Reason for exclusion
Gupta et al. [52] 2005	To compare a modified cardiac risk assessment to patients who died versus a group that survived	Death	Retrospective case control (Newcastle Upon Tyne, United Kingdom)	*n* = 247 146 cases (death) versus 101 control scores	Charts reviewed from 1996 to 2003	Modified Cardiac Risk Assessment (based on guidelines for perioperative CV evaluation for noncardiac surgery from the ACC/AHA task force)	Deceased group had higher CV risk scores. Correlation between risk score and mortality.	Case-control study design did not meet inclusion criteria; methodological issues

de Pádua Netto et al. [53] 2012	To assess ability of FRS to predict CV events in a population theoretically without risk factors	Unclear	Retrospective cohort study (Brazil)	*n* = 126 RTR with a functioning graft for >6 months	45 ± 16 years 65% male (*n* = 82)	Framingham Charts reviewed from 2005 to 2010	FRS does not adequately quantify real CV risk	Observational only; FRS in this population was assessed, but outcome was poorly described with no clear process defined to meet objective

CV: cardiovascular; ACC: American College of Cardiology; AHA: American Heart Association.

**Table 4:** Metrics of model performance and evaluation of bias.

Study	Model performance					Bias*					
	Discrimination	Calibration reported	Model fit reported	Reclassification reported	Analysis	Study participation	Study attrition	Prognostic factor measurement	Outcome measurement	Confounding measurement and account	Analysis
Kasiske et al. [25] 2000	No	No	No	No	Cox PH	Unsure	Unsure	Yes	Yes	yes	yes
Ducloux et al. [10] 2004	No	No	No	No	Cox PH (no continuous FHS risk)	Unsure	Unsure	Unsure	Yes	yes	no
Kiberd and Panek [28] 2008	Yes	Yes	No	No	ROC	Yes	No	Unsure	Unsure	yes	yes
Silver et al. [11] 2011	No	No	No	No	Cox PH (no continuous FHS risk)	Yes	No	Partially	No	Partially	yes
Israni et al. [33] 2010	Yes	Yes	Yes	No	Cox PH/KM	Unsure	Unsure	Unsure	Yes	yes	yes
Soveri et al. [21] 2012	Yes	Yes	No	No	Cox Ph	Yes	Unsure	Unsure	Yes	yes	yes
Soveri et al. [35] 2013	Yes	Yes	NA	No		Yes	Unsure	No	Yes	NA	NA

*Table 1 describes the criteria for bias assessment. Yes: adequately meets requirements for bias assessment (low risk of bias). No: does not adequately meet the requirements for bias assessment (high risk of bias). Partially: the study does address the component, but not in a satisfactory manner. Unsure: the authors did not make definitive statements to meet the requirements, but they are not necessarily absent from the study itself.
