# Supplementary material for: Validity of Cardiovascular Risk Prediction Models in Kidney Transplant Recipients
Source: ScientificWorldJournal. 2014 Apr 8;2014:750579. doi: 10.1155/2014/750579 (PMC3996891; doi:10.1155/2014/750579)
Supplement: Supplementary file 1 — Five databases were searched for this systematic review, including MEDLINE via OVID SP (1950 to present with daily update), EMBASE via OVID SP (1947 to present with daily update), CIHNAL via EBSCO, SCOPUS, and Web of Science (1900 to present). The complete search strategy for MEDLINE is listed below. Search strategies were modified appropriately with the assistance of a medical librarian for EMBASE, SCOPUS, and Web of Science. [file 750579.f1.docx]

Appendix A

Full search strategy for cardiovascular risk prediction models in renal transplant recipient reviews for MEDLINE. Search strategies were modified appropriately with the assistance of a medical librarian for EMBASE, SCOPUS, and Web of Science.

1 exp Cardiovascular Diseases

2 renal replacement therapy/ or kidney transplantation/

3 risk assessment/

4 framingham.mp.

5 procam.mp

6 “prospective cardiovascular munster”.mp

7 “assign(2W)scor*”.mp

8 “assign scor*”.mp

9 qrisk*.mp

10 scor.mp

11 “reynolds score” or “reynolds risk”

12 “risk scor*”.mp

13 “prediction rule”.mp

14 3 or 4 or 5 or 6 or 7 or 8 or 9 or 10 or 11 or 12 or 13

15 1 and 2 and 14
